# Supplementary material for: Paraneoplastic Resolution Holds Prognostic Utility in Patients with Metastatic Renal Cell Carcinoma
Source: Cancers (Basel). 2024 Oct 30;16(21):3678. doi: 10.3390/cancers16213678 (PMC11545392; doi:10.3390/cancers16213678)
Supplement: Supplementary file 1 [file cancers-16-03678-s001.zip › cancers-3257286-supplementary/Supplemental Table S1_ Lab References - Sheet1.pdf]

List of Paraneoplastic Syndrome	Institutional Normal Range for Test	Cutoff Used
**Anemia**		
Low Hemoglobin	M: 12.9-16.1 g/dL<br>F: 11.4-14.4 g/dL	<11.0 g/dL
Low Hematocrit	M: 37.7-46.5%<br>F: 33.3-41.4%	<33%
**Polycythemia**	M: 37.7-46.5%<br>F: 33.3-41.4%	M: Hct >52%<br>F: Hct >47%
**Thrombocytopenia**	150-400/nl	Platelet count <100/nl
**Thrombocytosis**	150-400/nl	Platelet count >400/nl
**Elevated CRP**	<10.0 mg/L	>5 mg/L
**Elevated ESR**	<30 mm/hr	M: >22 mm/hr<br>F: >29 mm/hr
**Hepatic Dysfunction**	ALT: 7-52 U/L<br>AST: 13-39 U/L	ALT >50 U/L<br>AST >50 U/L
**Hypercalcemia**	8.6-10.3 mg/dL	Corrected calcium >10.4 mg/dL
**Hyperuricemia**	2.3-7.6 mg/dL	M: >7 mg/dL<br>F: >5.7 mg/dL
**Elevated NLR**	N/A	>4:1 ratio

Abbreviations: renal cell carcinoma (RCC); paraneoplastic syndrome (PNS); male (M); female (F); hematocrit (Hct); alanine aminotransferase (ALT); aspartate aminotransferase (AST); C-reactive protein (CRP); estimated sedimentation rate (ESR); neutrophil-to-lymphocyte count.
