# Supplementary figures and images for: Paraneoplastic Resolution Holds Prognostic Utility in Patients with Metastatic Renal Cell Carcinoma
Source: Cancers (Basel). 2024 Oct 30;16(21):3678. doi: 10.3390/cancers16213678 (PMC11545392; doi:10.3390/cancers16213678)

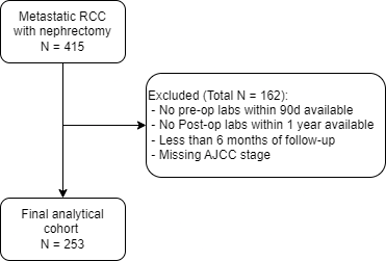

Supplement: Supplementary file 1 [file cancers-16-03678-s001.zip › cancers-3257286-supplementary/Supplementary Figure S1.png]
